# Dr. Thomas Wright Hall

**Published:** 1911-09-09

**Authors:** 


Dr. Thomas Wright Hall, M.D.Edin., has died at Ealing, where he was living in retirement. He was for many years in practice in Brazil as medical officer to the English staff of the Bahia and San Francisco Railway. He was a graduate of the University of Edinburgh, and qualified in 1853.

